# The Hippocampus: A Manifesto for Change

**DOI:** 10.1037/a0033650

**Published:** 2013-07-15

**Authors:** Eleanor A. Maguire, Sinéad L. Mullally

**Affiliations:** 1 Wellcome Trust Centre for Neuroimaging, Institute of Neurology, University College London, London, United Kingdom

**Keywords:** hippocampus, scene construction, space, episodic memory, amnesia

## Abstract

We currently lack a unified and mechanistic account of how the hippocampus supports a range of disparate cognitive functions that includes episodic memory, imagining the future, and spatial navigation. Here, we argue that in order to leverage this long-standing issue, traditional notions regarding the architecture of memory should be eschewed. Instead, we invoke the idea that scenes are central to hippocampal information processing. This view is motivated by mounting evidence that the hippocampus is constantly constructing spatially coherent scenes, automatically anticipating and synthesizing representations of the world beyond the immediate sensorium. By characterizing the precise relationship between scenes and the hippocampus, we believe a theoretically enriched understanding of its fundamental role and its breakdown in pathology can emerge.

Two issues are often conflated: how do we learn and remember our past experiences (episodic memory), and what does the hippocampus do. Despite the hippocampus being widely regarded as the quintessential episodic memory device, memory and the hippocampus are not simply interchangeable. Episodic memory is so much more than the hippocampus. We know this because episodic memory subsumes multiple cognitive processes, some of which are not hippocampal dependent (Nyberg, Kim, Habib, Levine, & Tulving, 2010). The hippocampus is also so much more than episodic memory. Although this has long been recognized in the animal literature (O’Keefe & Nadel, 1978), it has become increasingly apparent over the past two decades that the human hippocampus is also vital for a variety of cognitive functions that are not purely mnemonic, including spatial navigation (Spiers & Maguire, 2006), but also imagining fictitious (Hassabis, Kumaran, & Maguire, 2007; Hassabis, Kumaran, Vann, & Maguire, 2007) and future experiences (Addis, Wong, & Schacter, 2007; Hassabis, Kumaran, Vann, & Maguire, 2007; Schacter & Addis, 2009). To truly ascertain, then, what it is the hippocampus does, a useful strategy may be to consider the range of disparate cognitive functions that have been linked to it and deduce from this what common underlying processes or mechanisms may be hippocampally mediated.

Motivated by recent neuropsychological and neuroimaging findings, what follows are our current thoughts about this issue, culminating in a hypothesis that describes what we think the hippocampus does. This piece is not intended to be a forensic comparison of hippocampal theories, or an exhaustive literature review. We acknowledge that what we propose is not bulletproof and requires a good deal more investigation. Rather, our goal is to offer a fresh perspective, to stimulate new ways of thinking about the hippocampus. It is 56 years since formal cognitive studies of the human hippocampus began in earnest (Scoville & Milner, 1957), and yet we remain bereft of a widely agreed-upon answer to what it does. Our point here is that now may be the time to start asking different questions.

## Episodic Memory and Imagination

Two cognitive functions that have received much scrutiny in recent years are recollecting episodes from the past and imagining experiences that have not actually occurred. Whereas the former has a strong mnemonic context, the latter, residing in the realm of the imagination, seems to have less to do with memory. The finding that patients with bilateral hippocampal lesions have profound deficits on both tasks (Andelman, Hoofien, Goldberg, Aizenstein, & Neufeld, 2010; Hassabis, Kumaran, Vann, & Maguire, 2007; Klein, Loftus, & Kihlstrom, 2002; Mullally, Intraub, & Maguire, 2012; Race, Keane, & Verfaellie, 2011; Rosenbaum, Gilboa, Levine, Winocur, & Moscovitch, 2009; Tulving, 1985) therefore offered a novel avenue down which to explore the function of the human hippocampus.

**Figure 1 fig1:**
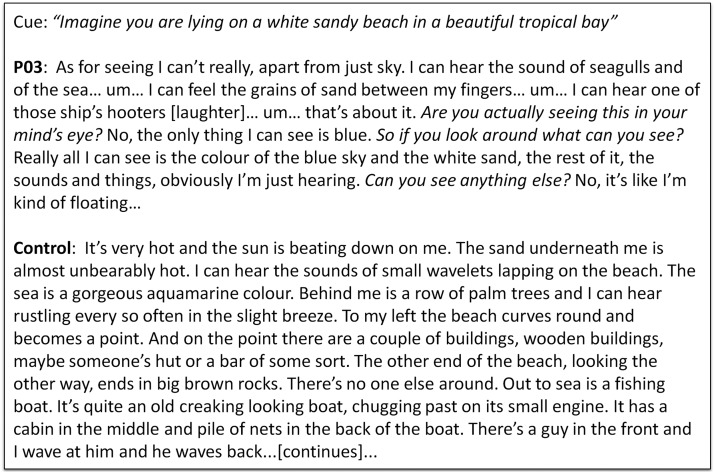
Examples of imagined scenarios. The cue is shown at the top, below which is an excerpt from P03, a patient with bilateral hippocampal damage, followed by that of a matched control participant. Reprinted from “Patients With Hippocampal Amnesia Cannot Imagine New Experiences,” by D. Hassabis, D. Kumaran, S. D. Vann, and E. A. Maguire, 2007, *Proceedings of the National Academy of Sciences of the United States of America, 104*, p. 1727. Copyright 2007 by the National Academy of Sciences.

One process that episodic memory and imagining future events seem to share is a subjective sense of the self over time (“autonoesis”; Tulving, 2002), which is closely coupled with the capacity for mental time travel (the ability to project oneself forwards or backwards in time). If the hippocampus is critical for this function, then it is not surprising that damage to the hippocampus impairs both episodic memory and future thinking (Buckner & Carroll, 2007). This hypothesis accommodates the neuropsychological profiles of patients like K.C. (Tulving, 1985) and D.B. (Klein et al., 2002), who were unable to recall their personal past or to imagine their own future. However, it does not account for why patients with hippocampal amnesia cannot construct spatially coherent atemporal fictitious scenes (see Figure 1), a task that is not future oriented or personally relevant (Hassabis, Kumaran, Vann, & Maguire, 2007; Mullally, Intraub, & Maguire, 2012).[Fig-anchor fig1]

Couching these patients’ deficits in terms of mental time travel and self-projection therefore seems insufficient. Indeed, a recent functional magnetic resonance imaging (fMRI) study revealed that frontal and parietal cortices, but not the hippocampus, supported mental time travel (Nyberg et al., 2010). Andrews-Hanna, Reidler, Sepulcre, Poulin, and Buckner (2010) showed that imagining scenes actually accounted best for activity in the hippocampus and medial temporal lobes, whereas other regions were concerned with the self and time. Overall, it seems that although the conscious awareness of time could be a core component of episodic memory, and the hippocampus might make use of temporal information when pertinent to the task at hand (MacDonald, Lepage, Eden, & Eichenbaum, 2011), the evidence suggests that for the hippocampus, it may not be primarily about time (Hassabis & Maguire, 2007).

**Figure 2 fig2:**
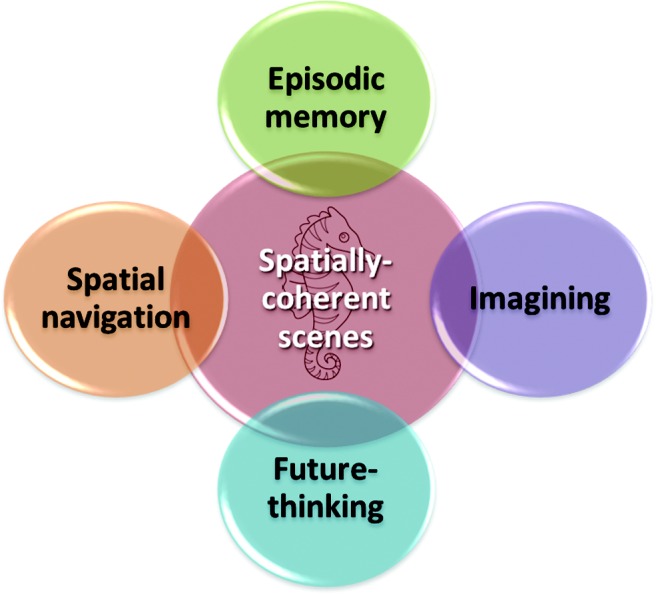
Scene construction theory contends that episodic memory, navigation, imagining fictitious scenes, and imagining the future encompass many processes that are not the primary concern of the hippocampus. Nevertheless, it proposes that they each rely on the hippocampus for a critical component, which is the construction of spatially coherent scenes.

The scene construction deficit observed in patients with bilateral hippocampal damage (Andelman et al., 2010; Hassabis, Kumaran, Vann, & Maguire, 2007; Mullally, Intraub, & Maguire, 2012; Race et al., 2011; Rosenbaum et al., 2009; Tulving, 1985), coupled with reports of selective scene-processing deficits following hippocampal damage (reviewed in Lee, Yeung, & Barense, 2012) and the fMRI observations that the healthy hippocampus is engaged when imagining fictitious (Hassabis, Kumaran, & Maguire, 2007) and future (Addis et al., 2007) scenes, led Hassabis and Maguire (2007, 2009) to propose the scene construction theory (SCT). The SCT posits that the hippocampus facilitates the construction of atemporal scenes allowing the event details of episodic memories and imagined future experiences to be martialed, bound and played out in a coherent spatial context. In this way, scene construction is held to underpin not only episodic memory and imagining the future but also cognitive functions such as spatial navigation (see Figure 2), and perhaps even mind wandering and dreaming. Placing scenes at the center of hippocampal information processing has intuitive appeal. For most people, recalling the past, thinking about the future, and planning how to get somewhere typically involves imagining scenes. Scenes are also a highly efficient way of packaging information.[Fig-anchor fig2]

The suggestion is not that the hippocampus alone is wholly responsible for all of these cognitive functions, but rather SCT proposes that despite the differences between them, these functions each require a crucial ingredient that is supplied by the hippocampus, and that is the ability to internally construct spatially coherent scenes. This resonates with the patients’ experiences of trying to imagine scenes:

There is no scene in front of me here. It’s frustrating because I feel like there should be. I feel like I’m listening to the radio instead of watching it on the TV. I’m trying to imagine different things happening, but there’s no visual scene opening out in front of me. (Mullally, Intraub, & Maguire, 2012, p. 266)

It’s hard trying to get the space, it keeps getting squashed. (Mullally, Intraub, & Maguire, 2012, p. 266).

Is this inability to imagine scenes responsible for the failure of both episodic memory and episodic-like imagination, or can existing theoretical frameworks account for these findings?

## Scene Construction and Relational Memory

An alternative explanation for these results is the constructive episodic simulation hypothesis (reviewed in Schacter et al., 2012). This theory proposes that simulation-related processes such as episodic memory, future thinking, and scene construction all require the extraction of relevant episodic details from memory and the recombination of these details to form fictitious simulations of novel scenes or events, and it is these processes that depend on the integrity of the hippocampus. In a similar vein, the relational theory proposes that the hippocampus is required for binding arbitrary or accidentally occurring relations among individual elements within an experience (Cohen & Eichenbaum, 1993; Eichenbaum & Cohen, 2001; Konkel & Cohen, 2009). By this account, one could argue that the patients failed to generate novel scenes because they were unable to bind together the disparate elements of the fictitious scene into a coherent whole, or they could not bind the items to the specific scene context (the binding of items and contexts model, reviewed in Ranganath, 2010).

These theories deny that there is anything special about the role of the hippocampus in the imagination of scenes, other than in constructing a scene, you must first retrieve the discrete scene elements from memory (constructive episodic simulation hypothesis) and bind them to one another and/or to the scene context (relational theory; binding of items and contexts model; constructive episodic simulation hypothesis). But is it possible, as asserted by SCT, that the hippocampus is engaged with scenes over and above binding items together or to a context? Evidence has emerged recently that suggests this may be the case (Mullally, Intraub, & Maguire, 2012).

## Scene Construction and Boundary Extension

**Figure 3 fig3:**
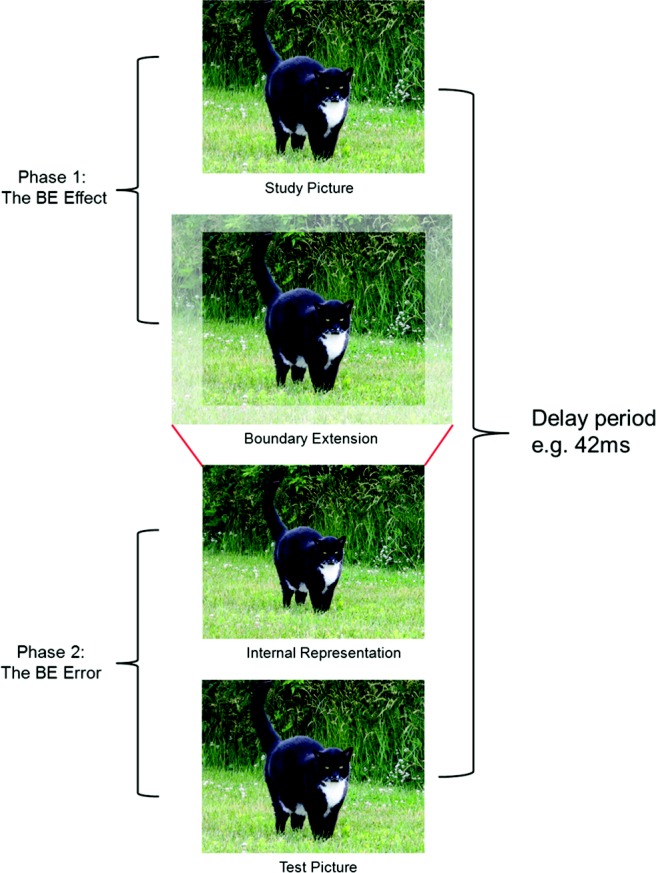
The phenomenon of boundary extension (BE). When we see a picture of a scene (top panel), we automatically extrapolate beyond the physical edges of that scene (second panel). This active extension of the scene is the “BE effect” (Phase 1). In Phase 2, when exactly the same picture is presented at test (fourth panel), we compare the now-extended internal representation of the original scene (third panel) to the test picture, leading to the impression that the test picture is “closer” than the original study picture, even though they are identical. This memory error is the “BE error.” From “The Hippocampus Extrapolates Beyond the View in Scenes: An fMRI Study of Boundary Extension,” By M. J. Chadwick, S. L. Mullally, and E. A. Maguire, in press, *Cortex*. Copyright by Elsevier. Reprinted with permission.

These new insights have arisen by bringing to bear a phenomenon not usually linked to the hippocampus, namely, boundary extension (BE; Intraub & Richardson, 1989). BE is where people remember seeing more of a scene than was present in the physical input, because they extrapolate beyond the borders of the original stimulus (see Figure 3). It is a robust and consistent effect found in adults (Intraub & Richardson, 1989; Seamon, Schlegel, Hiester, Landau, & Blumenthal, 2002), children (Seamon et al., 2002), and even babies (Quinn & Intraub, 2007). Of note, it only occurs in relation to scenes and not single isolated objects (Gottesman & Intraub, 2002). Interestingly, this dissociation mirrors the imagination profile observed in amnesic patients, where they are able to imagine single isolated objects, but cannot imagine scenes (Hassabis, Kumaran, Vann, & Maguire, 2007). BE is composed of two stages (see Figure 3). The first (the BE effect) requires intact scene construction because it involves the active extrapolation of the scene beyond its physical boundaries resulting in an internally generated “extended scene” representation, which persists when the scene is no longer visible. The second phase (the BE error) occurs at retrieval when this internally generated extended scene is conflated with the previously viewed scene from Phase 1, producing a memory error. Thus, when people are presented consecutively with exactly the same scene, they consistently judge the scene that is viewed second as being closer-up than the first scene, even though the two scenes are identical. The original scene need only be absent for as little as 42 ms for BE to be apparent, underscoring the online and spontaneous nature of the BE effect (Intraub & Dickinson, 2008).[Fig-anchor fig3]

Hippocampal-damaged patients’ faulty scene construction should impair their ability to extrapolate beyond the borders of the scene in Phase 1, and consequently they should fail to commit the BE memory error in Phase 2. This means that amnesic patients should display superior performance relative to healthy controls on the subsequent memory task. This paradoxical finding would be notable for two reasons. First, it would allow the patients’ scene construction and mnemonic problems to be disentangled. Second, it would facilitate exploration of whether the patients’ scene construction deficits are apparent when implicit scene construction is required as opposed to effortfully binding together disparate scene elements, as in the usual scene construction task.

**Figure 4 fig4:**
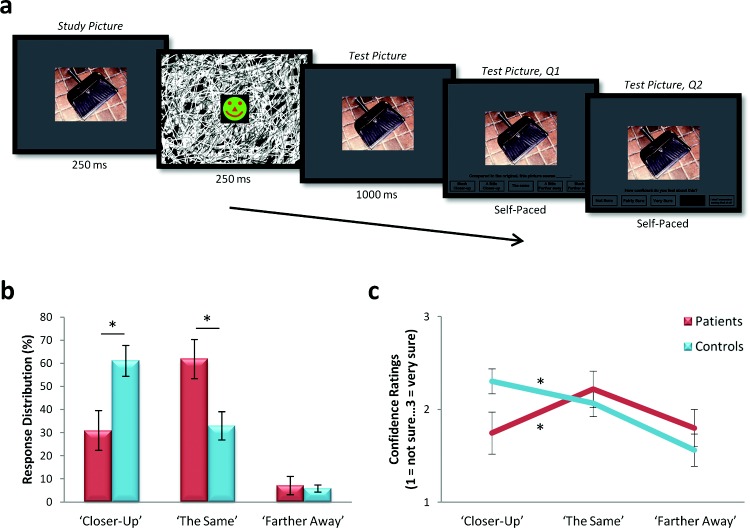
Rapid serial visual presentation boundary extension (BE) task. (a) Timeline of an example trial. After viewing two consecutively presented identical scenes, participants rated the perspective of the second scene relative to the first (as “closer-up,” “the same,” or “farther away”). (b) BE is revealed by a disproportionally large number of erroneous “closer-up” responses, which were greater for the controls than the hippocampal amnesic patients, who made significantly more accurate (“the same”) responses. (c) The control participants were significantly more confident when making their erroneous “closer-up” responses, whereas the patients were significantly more confident when making their correct “the same” responses. Means (±*SEM*). Q1 = Question 1; Q2 = Question 2. * *p* < .05. From “Attenuated Boundary Extension Produces a Paradoxical Memory Advantage in Amnesic Patients,” by S. L. Mullally, H. Intraub, and E. A. Maguire, 2012, *Current Biology, 22*, p. 263. Copyright 2012 by Elsevier. Reprinted with permission.

Mullally, Intraub, and Maguire (2012) investigated BE using the rapid serial visual presentation BE task alluded to above (see also Figure 4a) whereby participants were consecutively presented with two identical scenes and asked to rate the second scene relative to the first (note that on any one trial, the two scenes were identical). Seven patients with selective bilateral hippocampal damage and amnesia correctly identified that the study and test pictures were identical with greater frequency than controls, demonstrating more veridical memory (see Figure 4b). They also made significantly fewer BE-driven errors (“closer-up” responses), whereas the number of random errors (“farther away” responses) did not differ between the groups. Importantly, the patients displayed greater confidence in their correct “the same” judgments than in their incorrect (BE-driven) closer-up responses (see Figure 4c).[Fig-anchor fig4]

**Figure 5 fig5:**
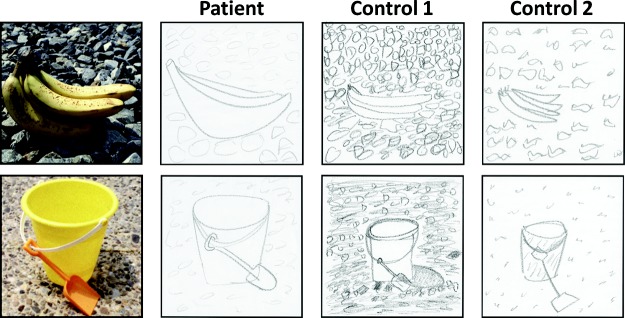
A drawing boundary extension (BE) task. The left panel shows two example scene stimuli that were studied for 15 s and immediately drawn from memory. The example control participants (middle and right panels) drew a more extended expanse of background than was present in the original stimuli (demonstrating BE). By contrast, the example patient’s drawings (left panel) are more accurate, showing a paradoxically better memory for the studied scenes. Reprinted from “Attenuated Boundary Extension Produces a Paradoxical Memory Advantage in Amnesic Patients,” by S. L. Mullally, H. Intraub, and E. A. Maguire, 2012, *Current Biology, 22*, p. 264. Copyright 2012 by Elsevier. Reprinted with permission.

Participants also performed other BE tasks in which they drew simple scenes from memory (drawing task; see Figure 5) and explored and reconstructed scenes while blindfolded using touch alone (haptic task). In both instances, the amnesic patients’ BE was greatly attenuated. These additional tasks highlight the accuracy with which the patients recollected the stimuli across short delays. For instance, in the haptic task, participants were required to study by touch alone a scene contained within four borders. At test, the scene borders were removed, and participants pointed to where each border had previously been positioned. Although the margins for error here were large, the patients’ placement of the borders was so accurate that the area of their reconstructed scenes did not differ significantly from the area of the studied scenes. This was not the case for controls, whose reconstructed scenes were significantly larger than the studied scenes. Overall, therefore, these hippocampal-damaged patients had significantly reduced BE relative to healthy-matched controls across three independent measures. However, being unencumbered by the BE effect meant that these amnesic patients displayed superior memory to that of non-amnesic controls.[Fig-anchor fig5]

Further direct evidence that these patients could not construct or visualize extended scenes came from a scene probe task. Participants were presented with a picture of a scene and asked to first extend this scene in their mind’s eye and, second, to describe what this extended scene would look like (Mullally, Intraub, & Maguire, 2012). Although the patients could accurately recount what they expected would be beyond the given view with ample detail, giving appropriate conceptual and contextual responses, their descriptions of the extended scene had significantly fewer spatial details (such as “on the right,” “in the distance”) than controls. Furthermore, when asked whether they were actually able to visualize this extended scene in their mind’s eye, the patients typically replied no. This deficit in the visualization of extended scenes reinforces the hippocampus’ role in the construction of spatially coherent scenes that are not physically in view.

Mnemonic accounts (Eichenbaum & Cohen, 2001; Ranganath, 2010; Schacter et al., 2012) of scene construction are therefore difficult to reconcile with the BE and scene probe data, as they would contend (a) that the patients performed differently to controls because they failed to encode or recollect the studied material, an explanation clearly incompatible with the BE findings; (b) the patients were unable to retrieve relevant constituent elements bound to the wider scene context, which was also clearly not the case (the scene probe data); and (c) the relational demands of the tasks precluded the patients performing in a normal fashion. However, the relational component of visualizing an extended scene is minimal and should be no greater in the spatial than in the conceptual domain (scene probe data). Moreover, new evidence is beginning to emerge suggesting that hippocampal-damaged patients can engage in counterfactual thinking (Mullally & Maguire, 2013). The patients demonstrated high-level reasoning ability, could deconstruct reality, add in and recombine elements, and change relations between temporal sequences of events, enabling them to mentally simulate plausible alternatives of complex episodes. However, counterfactual simulations that required the construction of an internal spatial representation were avoided by the patients. These emerging findings add weight to the view that the hippocampus is critical when there is a need to internally construct a coherent spatial scene within which to play out scenarios.

**Figure 6 fig6:**
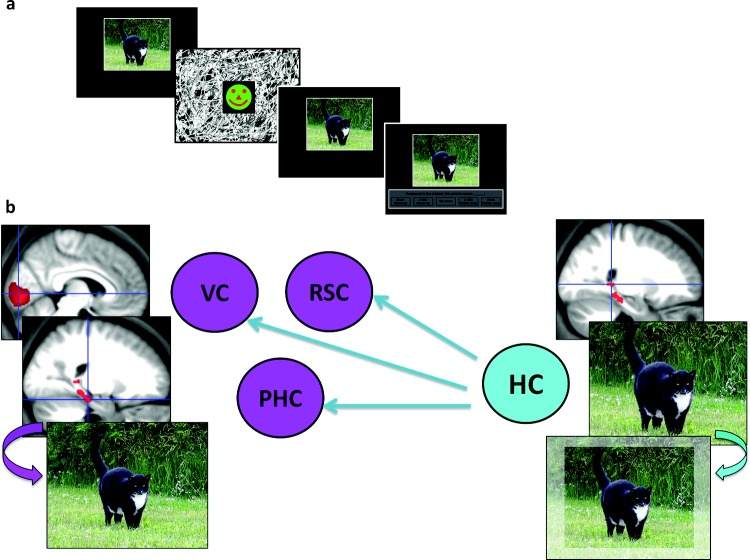
An functional magnetic resonance imaging study of boundary extension (BE; Chadwick et al., in press). (a) A modified version of the task described in Figure 4 was used with healthy participants. The analysis focused exclusively on the presentation of the first scene and contrasted trials in which the BE error was later committed with those where it was not committed. (b) This revealed activity in the hippocampus (HC) and parahippocampal cortex (PHC). Connectivity analyses showed that the HC seemed to drive the BE effect, exerting top-down influence on PHC, retrosplenial cortex (RSC), and as far back down the processing stream as early visual cortex (VC).

A recent fMRI study has thrown further light on BE and the role of the hippocampus. Chadwick, Mullally, and Maguire (in press) found that in healthy controls, the extrapolation of scenes (the BE effect) occurred rapidly around the time a scene was first viewed and was associated with engagement of the hippocampus and parahippocampal cortex (PHC; see Figure 6). Using connectivity analyses, they determined that the hippocampus drove the BE effect, exerting top-down influence on PHC and as far back down the processing stream as early visual cortex. These cortical regions subsequently displayed activity profiles that tracked the trial-by-trial subjective perception of scenes, rather than the physical reality, thereby reflecting the behavioral expression of the BE error. Thus, the hippocampus seems to directly influence our perceptual experience of the world through the implicit extrapolation of every scene we experience, a role not anticipated or explained by purely mnemonic (or temporal or simulation-based) accounts of hippocampal function. As such, the hippocampus and scenes may have an even more pervasive influence on our experience of the world than hitherto appreciated.[Fig-anchor fig6]

## Current Exposition of the SCT

These new findings (Chadwick et al., in press; Mullally, Intraub, & Maguire, 2012) show that the hippocampus is not only involved in constructing internal representations of scenes when explicitly requested, or even when you recall a past experience, plan a route, or imagine the future; in fact, the hippocampus is constructing scenes *all* the time. It is automatically, implicitly, and online anticipating and constructing a representation of the world beyond any given view. Thus, the hippocampus, although obviously involved in processing what you see, excels particularly at representing what you cannot see by constructing unseen scenes. In this way, the hippocampus’ role in scene processing differs from the contributions of other so-called scene-selective brain regions like PHC, which is believed to represent the local visual scene (Epstein, 2008, 2011) or three-dimensional space (Mullally & Maguire, 2011; Zeidman, Mullally, Schwarzkopf, & Maguire, 2012), and retrosplenial cortex, which may play a specific role in representing the most stable landmarks within an environment (Auger, Mullally, & Maguire, 2012). Moreover, these findings imply that the hippocampus is far from the stupid structure some have suggested, simply encoding everything into consciousness obligatorily and unselectively, leaving other areas such as the prefrontal cortex to sort the wheat from the chaff (Moscovitch, 2008). Nor does it appear to be specifically concerned only with the conscious (declarative) expression of long-term memory (Squire, 1992; but see Hannula & Greene, 2012). Instead, we suggest the hippocampus drives this automatic, anticipatory scene construction process, the product of which is fed back down the processing stream, enabling other areas to express the subjective experience of a seamless and continuous reality. Patients with hippocampal damage may therefore have to deal not only with problems recalling the past and difficulties with spatial navigation and imagining fictitious and future scenes. They may in fact be restricted to what is in front of their eyes, deprived of a level of subjective continuity, unable to visualize what is outside of their immediate view, what is behind them, or what is just around the corner.

The implication of this view is that the primary role of the hippocampus is not mnemonic. This idea is not new; in fact, some have argued that the concept of a hippocampal memory system is actually harmful (Gaffan, 2001, 2002; Horel, 1978), proposing instead that the hippocampus is involved “only to the extent that spatial information is needed for some kinds of memory” (Gaffan, 2001, p. 9). As such, memory impairments in patient populations are viewed as a by-product of their spatial processing deficits. Although this rejection of a mnemonic function for the hippocampus is extreme, others have highlighted the role of the hippocampus in perception as well as memory. For example, the emergent memory account (Graham, Barense, & Lee, 2010) suggests that memory deficits arise following medial temporal lobe damage because regions therein aid the construction of complex conjunctive object (perirhinal cortex) and spatial (hippocampus) representations that are essential for higher order perception and consequently memory. This model is supported by a series of neuropsychological (Barense et al., 2012; Lee et al., 2006; Lee, Buckley, et al., 2005; Lee, Bussey, et al., 2005; Lee, Levi, Davies, Hodges, & Graham, 2007) and fMRI studies (Barense, Henson, Lee, & Graham, 2010; Lee, Brodersen, & Rudebeck, 2013), which have led to the suggestion that the hippocampus plays a role in complex spatial perception, and specifically in the discrimination of complex scenes (see a review in Lee et al., 2012).

These latter observations accord with SCT in proposing that it may be short-sighted to regard the hippocampus as purely mnemonic. Of note, SCT can account for the above findings if one considers the nature of the tasks used in the studies that appear to be sensitive to hippocampal damage. One of the paradigms often used by Lee and colleagues is the oddity task (e.g., Lee, Buckley, et al., 2005) whereby scene stimuli are presented at different viewing angles, and participants must identify the incongruent scene. In such tasks, successful performance depends on intact scene construction because participants must be able to locate each viewpoint (and therefore reject the odd one out) within an overarching unified scene whose unseen aspects must be internally generated. Thus, SCT, unlike most other hippocampal theories, would actually predict the non-mnemonic, “perceptual” deficits observed by Lee and colleagues. In a similar vein, SCT also predicts hippocampal involvement in any task that requires scene construction, including those assessing working memory (Ranganath & Blumenfeld, 2005).

To summarize, although SCT does not preclude the existence of other mechanisms within the hippocampus, such as those that permit the binding of mnemonic details within a specific context (Ranganath, 2010), it maintains that for the hippocampus, scenes are special. As far as we are aware, this makes SCT unique, as no other theory of hippocampal function has scenes at its core. The removal of memory as the key hippocampal process enables SCT to reconcile unexpected hippocampal findings (such as the BE data and perceptual deficits) within a unified model that also accommodates the traditional mnemonic deficits observed in hippocampal amnesia.

## The Hippocampus and Episodic Memory

In this vein, it is important to note that SCT does not deny the existence of severe episodic memory problems in patients who have suffered hippocampal damage. However, it does not, as alluded to, place memory at the epicenter of hippocampal function. Rather, it proposes that episodic memories are encoded and recollected within a scene template, a template that is automatically and implicitly provided by the hippocampus. In this way, episodic memory impairments (and similarly deficits in imagining the future and spatial navigation) can result from disruption to the underlying scene construction process. Interestingly, however, retaining a basic ability to construct scenes is not sufficient to prevent these severe memory problems. Patient P01 was first reported by Hassabis, Kumaran, Vann, and Maguire (2007) as the only one of five densely amnesic patients with bilateral hippocampal damage to have preserved scene construction ability, contrasting with the grave difficulties experienced by the four other amnesic patients. This patient’s dense amnesia coupled with his intact scene construction therefore presented a challenge for SCT given that scene construction is conceptualized as a foundation step within the episodic memory hierarchy (see Figure 2).

Further investigation of P01 using fMRI revealed that when constructing scenes, he engaged the remnant of his right hippocampus (Mullally, Hassabis, & Maguire, 2012), showing that in some hippocampal-damaged amnesic patients, scene construction may be supported by residual hippocampal function. This finding accords with SCT in linking the hippocampus with scene construction but is at odds with the view of Squire et al. (2010), who argued that “for P01 future imagining was not hippocampal-dependent” (p. 19047; see also Maguire & Hassabis, 2011). Importantly, the data of P01 also inform the relationship between scene construction and episodic memory.

It appears that P01’s intact scene construction was not sufficient to rescue his episodic memory. This emphasizes that episodic memory should not be regarded as comprising scene construction alone. Although SCT argues that the hippocampus is primarily concerned with constructing scenes, and this underpins episodic memory by providing the spatial backdrop within which an episodic memory is played out, SCT explicitly recognizes that episodic memory requires additional processes on top of this scene construction process (Hassabis & Maguire, 2007, 2009). One possibility is that these additional inputs (concerned with the self and time) are supported by an extended network of brain areas (including the parietal and frontal cortices; Nyberg et al., 2010) that interact with the basic scene construction process within the hippocampus, and it is these hippocampal–cortical links that have become disrupted in P01, adversely affecting his episodic memory.

Another possibility is that these additional processes are also computed within the hippocampus. In this way, observations such as hippocampal time cells (MacDonald et al., 2011) could be accommodated within SCT. P01’s constellation of deficits could be explicable in terms of the location of his functioning hippocampal tissue, which in this case was in the right posterior hippocampus—a region that may be especially important for scene construction, but less so for episodic memory processes (Addis, Pan, Vu, Laiser, & Schacter, 2009; Mullally, Hassabis, & Maguire, 2012). More evidence is required to adjudicate between these intra- and extrahippocampal hypotheses, and examining functional differentiation down the long axis of the hippocampus may be particularly relevant here. Regardless, however, SCT does not permit the possibility that impaired scene construction could coexist with preserved episodic memory (the opposite pattern to that observed in P01) because within the SCT framework, scene construction and episodic memory are not considered to be independent processes. Rather, scene construction is viewed as an essential component of episodic memory without which episodic memories could not be truly and vividly expressed. Identification of patients demonstrating this particular dissociation is one way in which SCT could be falsified.

## Scene Construction and Verbal Memory

A finding that is challenging for SCT, and indeed for other hippocampal theories (Bird, Bisby, & Burgess, 2012; Moscovitch, Nadel, Winocure, Gilboa, & Rosenbaum, 2006; O’Keefe & Nadel, 1978; Ranganath, 2010; Schacter & Addis, 2009), is why patients with hippocampal amnesia are usually impaired on verbally mediated memory tasks such as recalling short stories and word-pair associates. One possibility is that scenes allow us to collate a lot of information in a quick, coherent, and efficient manner, and so we automatically use scene imagery during encoding and retrieval. For instance, we might visualize the context in which the story is unfolding, or place the items described in the word pairs in a simple scene together. Although this is speculative, it is not dissimilar to the mnemonic techniques, such as the method of loci, introduced in ancient Roman and Greek rhetorical treatises, in which memory for items is aided by placing them in specific physical locations. Despite the rise and fall of imagery-based memory theories across the decades (Paivio, 1969), there is much evidence to suggest that visual imagery not only boosts paired-associate recall in healthy participants but also enabled patients with left temporal lobectomies to partially compensate for their verbal memory deficits (Jones, 1974). If verbal memory tasks benefit from the use of imagery-based mnemonic strategies, and if these strategies involve scenes, then hippocampal patients would be disadvantaged on such tasks because of their scene construction problems. Alternatively, it could be that the neural processes supporting scene construction are also suited to coupling simpler stimuli together (such as two unrelated words), and in this way, hippocampal mechanisms originally developed to support scene construction and episodic memory have further evolved to support processing of non-scene stimuli. We suggest that exploring, in a wide-ranging and systematic fashion, the exact strategies people use when performing verbally mediated tasks may help in elucidating this important issue, which is one not discussed enough by extant hippocampal theories.

## Scene Construction and the Cognitive Map Theory

The hippocampus has long been linked to spatial navigation, with place cells in the hippocampus exhibiting location-specific firing (O’Keefe & Dostrovsky, 1971). This led to the cognitive map theory, which posits that a fundamental function of the hippocampus is the construction and maintenance of allocentric spatial maps of the environment (O’Keefe & Nadel, 1978). This spatial role might also account for episodic memory deficits because personal memories always occur in a spatial context (Burgess, Maguire, & O’Keefe, 2002). SCT and cognitive map theory on the surface appear to have much in common. However, an important difference becomes obvious when considering the most recent incarnation of the cognitive map theory, the boundary vector cell (BVC) model (reviewed in Bird et al., 2012).

The BVC model proposes that hippocampal place cell firing (thresholded via inputs from BVC s that code the location of the rat relative to environmental boundaries) functions to enable an animal to form a representation of where it thinks it is in the environment. In this way, environmental boundaries are considered to be central to spatial cognition, aiding the encoding, retrieval, and imagination of spatial contexts. The findings that hippocampal activity appears to be correlated with the number of boundaries imagined during fMRI (Bird, Capponi, King, Doeller, & Burgess, 2010) and place cell activity becomes less spatially specific when boundaries are removed (Barry et al., 2006) appear to bolster this argument.

However, given the BE findings, SCT views physical environmental boundaries differently, arguing instead that a place cell’s response to the presence of boundaries could be due to the increased demand for it to compute what might be on the other side of the boundary (as this is now hidden from view). Similarly, the decrease in spatial specificity observed as a consequence of boundary removal may arise because these computations are no longer needed (see also Hayman, Donnett, & Jeffery, 2008, who reported a failure of place cells to be controlled by an acoustic, as opposed to a visually occluding, boundary). In this way, the BVC model views boundaries as inward bounding (containing the space within specific boundaries), whereas SCT views boundaries (either physical boundaries such as walls or the edges of our current view) as outward looking, the point from which the hippocampus must extrapolate in order to make predictions about, and so imagine, the upcoming spatial environment.

## Conclusions

The nub, then, of our proposal is that scenes are the primary currency of the hippocampus. For many of us, scenes are the language of thought, and we argue that the hippocampus actively and automatically predicts and constructs the scenes we need to fuel our cognition. Up until now, scenes have not featured prominently in accounts of hippocampal function. SCT, although not denying the important role of the hippocampus in memory, challenges us to consider how it accomplishes this, and urges consideration of the potential importance of scenes. The memory literature is awash with experiments investigating hippocampal processes (such as associative memory) that have used scene stimuli but that pay little or no attention to the effect of the scenes themselves. Recognizing the possibility that the scene stimuli could be influencing hippocampal function could alter the conclusions of such studies.

SCT’s perspective on the hippocampus not only offers a potentially unifying model for understanding the disparate roles of the hippocampus, but could facilitate a new hippocampal dialogue because, unlike episodic memory, scenes are amenable to testing in a range of species (Killian, Jutras, & Buffalo, 2012). The construction of scenes and BE involve anticipating what is likely to be beyond the view. This also resonates with emerging ideas concerning prior knowledge and schema (Tse et al., 2007), templates (Dragoi & Tonegawa, 2011), and predictive coding (Friston, 2010). Importantly, then, scene construction generates new questions and testable hypotheses that could provide fresh impetus to the field.

Clearly, this view requires much further investigation and nuancing, not least to elucidate the status of BE in patients with lesions (bilateral and unilateral) in areas other than the hippocampus, to uncover exactly how the hippocampus facilitates scene construction, to examine the precise relationship of scene construction with computations that occur in the hippocampus such as pattern separation and pattern completion (which may underpin the BE effect), and of course to definitively establish that an inability to construct scenes directly explains the full constellation of deficits that arise from hippocampal pathology. Nevertheless, by releasing the hippocampus from currently constrained accounts of its function, we believe that a theoretically enriched understanding of its fundamental role and its breakdown in pathology can emerge.
